# Gender-Affirming Hormone Therapy, Vascular Health and Cardiovascular Disease in Transgender Adults

**DOI:** 10.1161/HYPERTENSIONAHA.119.13080

**Published:** 2019-10-28

**Authors:** Paul J. Connelly, E. Marie Freel, Colin Perry, John Ewan, Rhian M. Touyz, Gemma Currie, Christian Delles

**Affiliations:** 1 From the Institute of Cardiovascular and Medical Sciences, British Heart Foundation, Glasgow Cardiovascular Research Center, University of Glasgow, United Kingdom (P.J.C., E.M.F., C.P., R.M.T., G.C., C.D.); 2 Sandyford Sexual Health Service, NHS Greater Glasgow and Clyde, Glasgow, United Kingdom (J.E.).

**Keywords:** cardiovascular, disease, diabetes mellitus, dyslipidemias, hypertension, stroke, thrombosis, transgender persons

## Abstract

Gender-affirming or cross-sex hormone therapy is integral to the management of transgender individuals yet our appreciation of the effects of such hormones on cardiovascular health is limited. Insights into vascular pathophysiology and outcomes in transgender people receiving sex steroids could be fundamental in providing better care for this population through the management of cardiovascular risk and more broadly advance our understanding of the role of sex and gender in vascular health and disease. In addition, there is a need to understand how gender-affirming hormone therapy impacts cardiovascular disease risk and events as transgender individuals age. This review explores the available evidence on the associations between gender-affirming hormones and cardiovascular events such as coronary artery disease, stroke, hypertension, thrombosis, lipid abnormalities, and diabetes mellitus. Current research about vascular outcomes in adults receiving hormonal therapy is limited by the absence of large cohort studies, lack of appropriate control populations, and inadequate data acquisition from gender identity services. Existing epidemiological data suggest that the use of estrogens in transgender females confers an increased risk of myocardial infarction and ischemic stroke. Conversely, transgender males receiving testosterone lack any consistent or convincing evidence of increased risk of cardiovascular or cerebrovascular disease. Further studies are required to confirm whether such risk exists and the mechanisms by which they occur.

Transgender people experience gender dysphoria due to incongruence between their gender identity and the sex they were assigned at birth (Table 1).^1^ Due to lack of accurate census data, barriers to healthcare such as social stigma and variable engagement with gender-affirming healthcare, the size of the transgender population is uncertain, however, it is estimated to represent ≈0.5% of the US population.^1–5^ An expansion in population prevalence and the utilization of transgender health services has been observed in recent years.^6–8^

Gender-affirming hormone therapy (GHT) aims to align the characteristics of an individual with their gender identity. The mainstay of this lifelong treatment in transgender males (TGM) is testosterone, typically delivered as intramuscular testosterone undecanoate or ester formulation. Transgender females (TGF) receive oral or transdermal estrogen preparations (eg, estradiol valerate or hemihydrate) often in conjunction with either a gonadotropin-releasing hormone analog (eg, Goserelin) or an anti-androgen (eg, cyproterone acetate).^9,10^

A deeper understanding of the alterations in vascular pathology and outcomes in transgender people receiving GHT could be fundamental in informing the management of cardiovascular risk. This review outlines the current evidence relating to the effects of GHT, and in particular exogenous testosterone and estrogen, on vascular health outcomes in transgender adults.

## Transgender Health

### Ischemic Heart Disease

The first observational study relating to GHT and cardiovascular disease (CVD) was published in 1989 (Table 2). This did not demonstrate any difference in the crude incidence of myocardial infarction (MI) or mortality relating to MI over a 4-year follow-up period when comparing 303 TGF to cisgender males (CGM). No cases of MI were noted within the TGM group.^11^

In 1997, van Kesteren et al^12^ undertook a retrospective observational study in 816 TGFs and 293 TGMs receiving GHT with a respective follow-up of 7734 and 2418 patient-years. This study demonstrated a decreased standardized incidence ratio (SIR) of MI in TGFs (SIR, 0.5 [95% CI, 0.24–0.91]) compared with CGMs. There was no difference in MI standardized mortality ratio (SMR, 0.71 [95% CI, 0.26–1.55]). SIR was no different in TGMs compared with cisgender females (CGFs).

In 966 TGFs and 365 TGMs with a median follow-up of 18.5 years, mortality was 51% higher compared with the general population.^13^ This was attributed to increased suicide, HIV infection, CVD, and substance abuse. TGFs had a SMR of 1.64 (95% CI, 1.43–1.87) relating to ischemic heart disease. The adjusted hazard ratio (HR) for cardiovascular mortality was 3.12 (95% CI, 1.28–7.63) in those using ethinylestradiol compared to former or never-users. TGM demonstrated a nonsignificant SMR of 1.19 (95% CI, 0.39–2.74) for ischemic heart disease mortality.

A population-based matched cohort study of 324 transgender people who had undergone gender reassignment surgery with mean follow-up of 11 years demonstrated an increased risk of cardiovascular death (HR, 2.5; 95% CI, 1.2–5.3) compared with cisgender people.^14^ In a cohort study of 100 transgender people retrospectively followed-up for 10 years, 2 cases of MI were identified in TGFs and no cases in TGM.^15^ A cross-sectional study in 138 TGM and 214 TGFs receiving GHT over an average of 7.7 years (range 4–13 years) demonstrated significantly increased prevalence of MI in TGFs (18.7 per 1000) compared with CGFs but not CGMs.^16^

Studies using data from the Behavioral Risk Factor Surveillance System showed that transgender individuals are at greater risk of MI than cisgender individuals.^17–19^ Meyer et al^17^ reported a higher odds ratio (OR) of MI in transgender (OR, 1.82 [95% CI, 1.22–2.72]) compared with cisgender people, with no increased risk of angina or coronary heart disease (OR, 1.37 [95% CI, 0.83–2.25]). Nokoff et al^18^ demonstrated an increased risk of MI (OR, 2.9 [95% CI, 1.6–5.3]) in TGFs compared with CGFs, but not CGMs (OR, 1.09 [95% CI, 0.59–2.03]). There was no increased risk comparing TGMs to CGMs or CGFs. The risk of angina or coronary heart disease was not increased in either TGFs or TGMs. Conversely, Alzahrani et al^19^ demonstrated that TGM but not TGF had an associated higher risk of MI compared with their natal sex comparators after adjusting for traditional CVD risk factors. Importantly these studies are cross-sectional, do not confirm the use of GHT and rely on the self-reporting of health issues such as MI and are, therefore, at risk of significant bias.

In a retrospective analysis of electronic medical records between 2006 and 2014, 2842 TGFs and 2118 TGMs receiving GHT were followed up for 4.0 and 3.6 years, respectively. A higher rate of MI in TGFs was observed compared with CGFs (HR, 1.8 [95% CI, 1.1–2.9]).^20^ However, this was not increased when compared with CGMs (HR, 0.9 [95% CI, 0.6–1.5]). Again, no differences were observed between TGMs, CGMs, or CGFs.

Overall, evidence derived from age-adjusted population comparisons and age-matched cohort studies suggest that TGFs have a higher risk of CVD and ischemic events than CGFs, however, in only one study was this higher than CGMs.^13^ Importantly, most studies use TGF populations under the age of 50 years, and little is known of the impact of GHT on vascular health in the longer term. As discussed in recent meta-analysis, there is a paucity of data about important patient outcomes, such as MI, and the ability to draw conclusion from studies where control groups and age-matched cohorts are not clearly defined impedes any meaningful assessment.^21^ No consensus exists on whether a transgender person’s cardiovascular risk should be compared with their natal sex or gender.

### Cerebrovascular Disease

In an observational study of 303 TGFs followed up for a median of 4.4 years, there were no differences in the crude incidence of transient ischemic attack compared with CGMs.^11^ Similar results were observed by van Kesteren et al,^12^ who did not demonstrate any significant difference in SIR for cerebrovascular disease in TGFs. In a study of 966 TGFs and 375 TGMs with a median follow-up of 18.5 years, there was no difference in mortality associated with stroke in TGFs and no cases at all in TGMs.^13^

More recently, Wierckx et al^16^ undertook a case-control study in 214 TGFs and 138 TGMs with an average of 7.7 years GHT and mean age of 43 years. TGFs demonstrated a higher prevalence of transient ischemic attack and cerebrovascular disease (23.4 per 1000), which, although no different from age-matched CGFs (14.9 per 1000), was significantly higher than age-matched CGMs (9.4 per 1000). No cases were observed in CGMs.

Survey data demonstrated no difference in cerebrovascular disease risk in transgender compared with cisgender populations.^17^ When stratified between TGFs and TGMs there was no increased risk in comparison to CGMs or CGF.^18^ The use of GHT was uncertain in these populations.

Getahun et al^20^ undertook an electronic medical record-based cohort study in 2842 TGFs and 2118 TGMs. TGFs had higher rates of ischemic stroke compared with corresponding rates in CGM and CGF. Ischemic stroke rates were highest in a sub-analysis of 853 TGFs who initiated GHT during the study period. These rates did not differ compared with cisgender populations during the first 6 years of follow-up but thereafter increased substantially compared with both CGMs (HR, 9.9 [95% CI, 3.0–33.1]) and CGFs (HR, 4.1 [95% CI, 1.5–11.4]).

Although uncertainty remains, TGFs appear to be at a higher risk of developing ischemic stroke compared with cisgender populations. This risk is most evident in longer-term estrogen use. Conversely, TGMs do not have increased cerebrovascular disease risk.

### Hypertension

Numerous studies have suggested an increased incidence of hypertension in transgender populations. Asscheman et al^11^ observed an increase in the crude incidence of hypertension in 303 TGFs. However, no increase was demonstrated in TGMs. The SIR for hypertension (defined as >160/95 mm Hg) was not significantly higher in TGFs or TGMs compared with CGMs and CGFs, respectively. In a cohort study comparing 63 untreated TGMs to 48 TGMs undergoing GHT, the resting systolic and diastolic brachial blood pressures (BPs) in the treated group were significantly higher.^22^ After 2 years of GHT in 79 TGFs and 43 TGMs, the mean of 3 consecutive systolic BPs increased significantly by 17.8 mm Hg and 13.4 mm Hg, respectively.^23^ The TGF group also demonstrated a 3.17 mm Hg increase in diastolic BP, which was not noted in the TGM group.^23^ Lastly, self-reported hypertension in 369 TGFs and 239 TGMs was no higher when compared with cisgender populations.^18^

Furthermore, prospective studies demonstrated alteration in BP following GHT initiation. In a study of 20 TGFs commencing GHT an increase in both mean systolic (7 mm Hg) and diastolic (5.7 mm Hg) BP (measured every 5 minutes for 2 hours using an automatic device) was noted at one year.^24^ No significant changes in BP were noted in 17 TGMs in this timeframe. Oral estradiol valerate has been shown to reduce systolic BP by 6.3 mm Hg after 1 year in 40 TGFs. This was not observed in a transdermal estrogen cohort (n=13) or in TGMs receiving testosterone undecanoate.^25^ TGMs treated with intramuscular testosterone have demonstrated a 3.2 mm Hg increase in systolic but not diastolic BP.^26^ In 2019, van Velzen et al^27^ reported a prospective observational study of 242 TGFs and 188 TGMs, where BP was measured via an automated device 12 months after commencing GHT. In TGMs, no change was observed in systolic BP. However, diastolic BP significantly increases by 3 mm Hg in the pooled analysis. In TGFs, in whom 59.5% used oral estrogen valerate and 40.5% were prescribed transdermal estrogen patches, systolic and diastolic BP decreased by 3 and 2 mm Hg, respectively.

Results from these studies are inconsistent, and alterations in BP may be dependent on factors such as GHT duration and age. GHT appears to elevate BP in both TGMs and TGFs in most studies. However, this may only be apparent with longer-term use.

### Venous Thromboembolism

GHT may also promote CVD by altering the thrombotic phenotype. Early observational studies demonstrated a 20 to 45 fold increase in the rates of venous thromboembolism (VTE) in GHT-exposed TGF.^11,12^ In a more recent retrospective analysis, VTE developed in 1.2% of TGF receiving oral therapy for 2 years and was more common in those exposed to conjugated equine estrogen (4%) compared with estrogen valerate (0.6%) or ethinylestradiol (0.7%).^28^ Subsequently, a case-control study of 214 TGFs with a mean estrogen duration of 7.7 years VTE occurred in 5.1%.^16^ The higher risk of VTE observed by Getahun increased with time, with 2- and 8- year risk differences of 4.1 (95% CI, 1.6–6.7) and 16.7 (95% CI, 6.4–27.5) per 1000 persons relative to CGM.^20^ Although the majority of studies demonstrate an increased risk in TGFs, this finding is not uniform.^29,30^ To our knowledge, no study has demonstrated an increased risk of VTE in TGMs.

### Cardiometabolic Risk

GHT may also alter cardiometabolic parameters through which it may modify CVD risk. In a recent systematic review and meta-analysis 29 studies, including 4731 transgender individuals (68% TGFs) with follow-up ranging from 3 months to 41 years, were assessed.^21^ Testosterone therapy in TGMs, at greater than 2 years follow up was associated with an elevation in triglycerides (21.4 mg/dL; 95% CI, 0.14–42.6) and LDL (low-density lipoprotein; 17.8 mg/dL; 95% CI, 3.5–32.1) with reductions in HDL (high-density lipoprotein; −8.5 mg/dL; 95% CI, −13 to −3.9) with no significant change in total serum cholesterol. Conversely, no significant differences were apparent in LDL, HDL, or total cholesterol in TGFs, but an increase in triglycerides was observed (31.9 mg/dL; 95% CI, 3.9–59.9). Although this assessment is limited by low-quality evidence, it raises questions as to why lipids do not reflect their adopted and more favorable gender profile in TGF and why this shift in TGM does not confer an increased risk of CVD.

It has also been suggested that an increased risk of type 2 diabetes mellitus may exist in this population. In an observational study of 966 TGFs receiving cyproterone acetate and either oral or transdermal estrogen, there was no significant difference in diabetes mellitus-related SMR compared with the cisgender population (SMR 0.85; 95% CI, 0.41–1.32), while no cases were identified in 365 TGMs.^13^ In a cross-sectional study completed in 214 TGFs and 138 TGMs, there were higher rates of diabetes mellitus in TGFs compared with CGFs or CGMs (42 per 1000 cases).^16^ TGMs demonstrated a higher incidence (36.2 per 1000 cases) compared with CGFs but not CGMs. Increases in homeostatic model assessment for insulin resistance index in TGFs has been demonstrated after 2 years of transdermal estrogen, but not after 1 year or in TGM.^23,31^ Fasting blood glucose in TGF is higher with transdermal estrogen but not oral ethinylestradiol.^23,24^ Studies using the self-reporting Behavioral Risk Factor Surveillance system with no GHT confirmation did not demonstrate a higher risk of diabetes mellitus.^17,18^ Consequently, the relationship between GHT and altered glucose metabolism remains uncertain.

### Surrogate Markers of Cardiovascular Risk in Transgender People

Few studies have assessed the effects of GHT on surrogate markers of cardiovascular risk in transgender people. TGFs prescribed either ethinylestradiol or conjugated estrogen for an average 61 months demonstrated improvements in flow-mediated and nitric oxide-induced vasodilation compared with cisgender individuals. No improvements in exercise-induced metabolic vasodilation were observed.^32^ Oral estrogen in 23 TGFs resulted in increased levels of IL (interleukin)-6, IL-1, and IL-8 in the first few months of GHT and fell thereafter, while levels of the anti-oxidant enzyme superoxide dismutase were elevated throughout the 6-month study period, which was not apparent in those prescribed transdermal estrogen.^33^ After 4 months of GHT endothelin levels increased in TGM and fell in TGF.^34^ Oral estrogen may also increase nitric oxide and decrease tissue plasminogen activator levels.^35,36^

Brachial-ankle pulse wave velocity was higher in GHT-exposed versus untreated TGM with no difference in carotid augmentation index.^22^ GHT in TGMs results in higher brachial artery diameter and reduced nitrate-induced vascular response, but similar endothelial function compared with that of age-matched controls.^37^ Reductions in estradiol in TGM treated with the aromatase inhibitor, anastrazole, in addition to parenteral testosterone esters, resulted in no change in flow-mediated dilation, however, a significant positive correlation in common carotid compliance and distensibility coefficients were observed.^38^ In 56 transgender individuals, carotid intima-media thickness and flow-mediated dilation of the brachial artery was measured 2 to 3 months after gender reassignment procedures and compared with those receiving GHT alone.^39^ Those undergoing surgery experience a reduction in flow-mediated dilation and increased carotid intima-media thickness.

Overall data describing the influence of GHT on vascular structure and physiology is limited. The GETS (Gender Dysphoria Treatment in Sweden) study primarily aims to assess the physiological and epigenetic effects of initiating GHT on skeletal muscle and adipose tissue, and if such changes influence metabolism and body composition.^40^ Secondary outcome measures include the assessment of carotid augmentation index, echocardiograms, coronary flow reserve, and carotid intima-media thickness.

## Sex Steroids and the Vasculature

**Figure 1.**
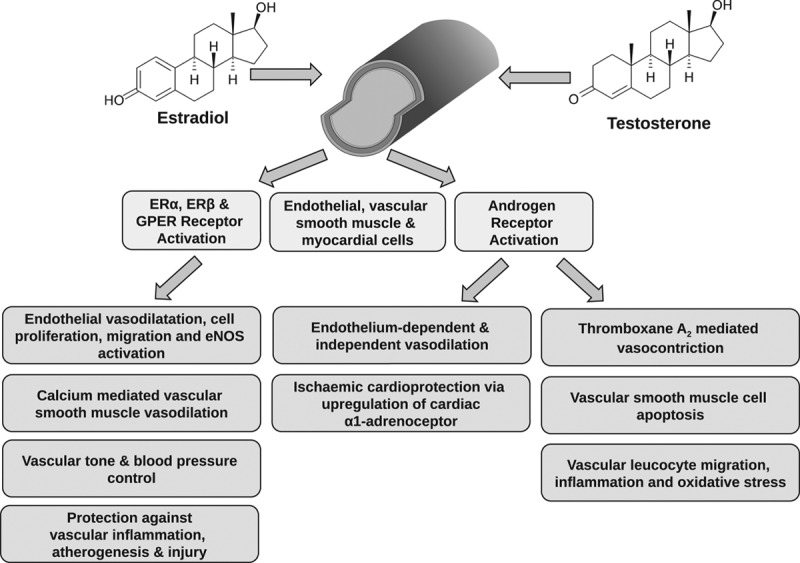
Vascular effects of sex steroids via estrogen and androgen receptors expressed in endothelial, vascular smooth muscle, and myocardial cells.

A compressive review of complexity of sex hormone receptor expression, signaling and regulation of vascular function is beyond the scope of this article. However, excellent reviews on this topic are available.^41–44^ In short, the vascular effects of estrogen are mediated via the estrogen receptors (ER-α, ER-β, and the GPER [G protein-coupled estrogen receptor]). These are expressed in endothelial, vascular smooth muscle, and myocardial cells (Figure 1). In mice, ER-α mediates protective effects of estrogen in response to vascular injury and atherosclerosis.^45,46^ In endothelial cells, ER-α has been shown to activate endothelium-dependent vasodilatation via endothelial nitric oxide synthase, endothelial proliferation, and migration, and promotes carotid artery re-endothelialization.^47,48^ Mouse models have also demonstrated an important role of ER-β in the regulation of vascular function and blood pressure ^49^ while loss of GPER action augments endothelium-dependent vasoconstriction and promotes atheroma formation and inflammation.^50^

**Figure 2.**
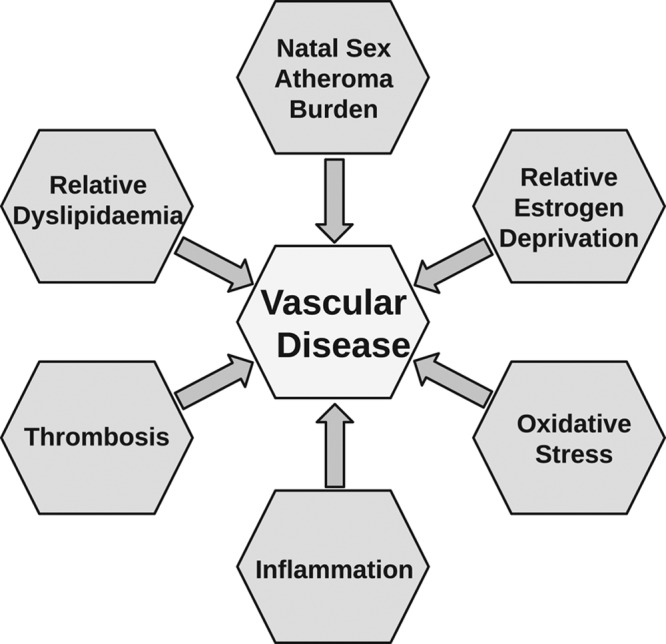
Putative model for increased vascular risk in transgender women.

A paradoxical relationship, therefore, exists between the observed role of estrogen in the vasculature and the clinical outcomes observed in TGF. In postmenopausal CGF, estrogen’s age and exposure-dependent vasoprotective effects suggest a beneficial role in early, but not later stages, of atherogenesis.^51–53^ Similarly, in apolipoprotein E-deficient mice, exogenous estrogen precludes atheroma development but not the progression of established lesions.^54^ In addition to the greater atheroma burden associated with their natal sex, TGFs are deprived of the protection of endogenous life-long estrogen exposure (Figure 2).^55^ Alterations to estrogen receptor subtype expression, which is sex and tissue-specific, receptor sensitivity or signaling may contribute to increases in oxidative stress and inflammation in this relatively dyslipidemic, pro-hypertensive, and pro-thrombotic phenotype.^33,56,57^ Mechanistic studies exploring this relationship have yet to be performed.

Much like ERs, the AR (androgen receptor) is expressed in vascular endothelial and smooth muscle cells.^44^ In vitro studies demonstrate that testosterone acts as a vasodilator via both endothelium-dependent and independent mechanisms.^58,59^ This action is mediated, in part, by inhibition of voltage-operated calcium channels and the activation of potassium channels on vascular smooth muscle cells.^60^ Conversely, vasoconstrictive properties of testosterone have been observed in isolated and perfused rat coronary arteries.^61^ Testosterone has been shown to confer cardioprotection after ischemia in isolated perfused hearts and ventricular myocytes from orchidectomized rats via upregulation of the cardiac α1-adrenoceptor.^62^ In rats, testosterone also induces leukocyte migration via NADPH oxidase cyclooxygenase-dependent mechanisms and may contribute to inflammatory processes and oxidative stress in the vasculature, thereby potentially increasing cardiovascular risk.^63^ Testosterone induces apoptosis in vascular smooth muscle cells via the extrinsic apoptotic pathway with the involvement of AR activation and mitochondria-generated reactive oxygen species.^64^

Consequently, testosterone has both vasoprotective and vasoinjurious actions. It is unclear why the latter does not precipitate cardiovascular risk in TGMs or if androgen deprivation is responsible for an increased cardiovascular risk in TGF. This is further complicated by the use of gonadotropin-releasing hormone agonists, which receptor is expressed in the cardiovascular system and may increase cardiovascular risk in the treatment of prostate cancer, albeit in the context of hypogonadism.^65,66^

## Conclusions

Sexual dimorphism exists in cisgender people across all spectrums of diseases including immune response, inflammatory disorders, malignancy, clinical pharmacology, and psychiatry.^67–71^ Although this review focuses on vascular disease, GHT impacts other aspects of health such as bone metabolism and the risk of malignancy. Therefore a cross-disciplinary approach is required to provide transgender people with optimal care.^9,10^

Although, GHT is integral to the management of transgender individuals the majority of studies identified by this review are retrospective and do not offer insight into the mechanisms by which sex steroids may alter vascular physiology and sex hormone receptor status in transgender people.^72,73^ The dose, formulation and mode of hormone therapy are heterogeneous, and achievement and sustainment of target hormone levels are often omitted. Their interpretation is also confounded by rates of mental health disorders, substance abuse, and health inequalities in transgender populations, which will contribute to the burden of cardiovascular risk.^74^ Furthermore, the long-term effects of pubertal suppression and subsequent introduction of GHT on vascular health in adolescents is unknown. Lastly, a consensus must be reached as to whether transgender individuals should be compared with their natal sex or their adopted gender.

Current evidence suggests that the use of estrogen by TGFs confers an increased risk of MI and ischemic stroke (Table 3). Whether this is a consequence of GHT or legacy effect of natal sex remains unclear as not all studies demonstrate increased risk compared with CGMs. Conversely, TGMs lack any consistent or convincing evidence of increased risk of cardiovascular or cerebrovascular disease despite blood pressure elevations and dyslipidemia. The study of cardiovascular health and disease in transgender people is urgently required to implement better clinical care and evidence-based guidance.

**Table 1.**
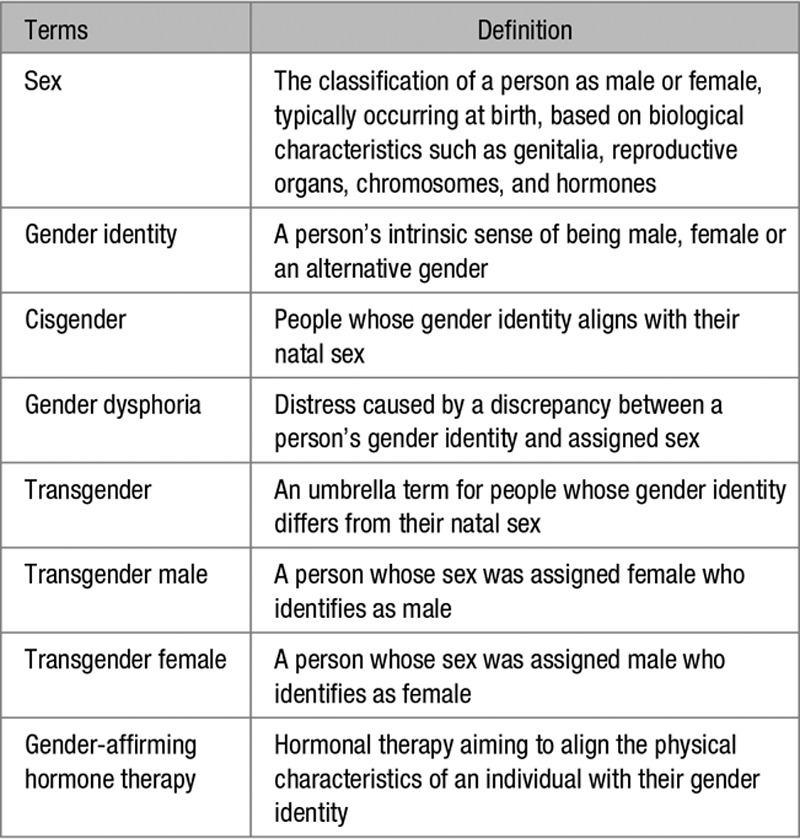
Terms Used in Transgender Medicine

**Table 2.**
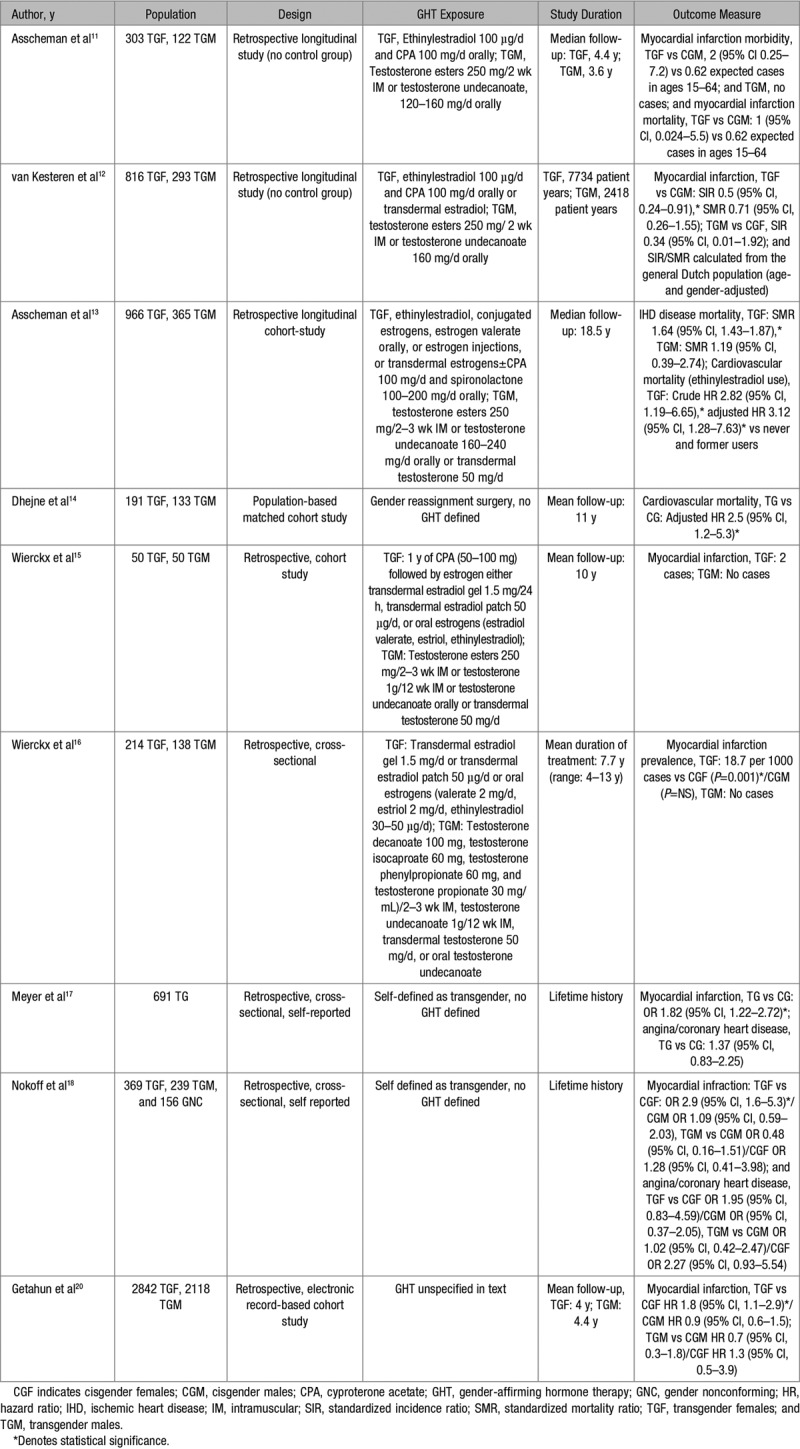
Ischemic Heart Disease in Transgender Populations

**Table 3.**
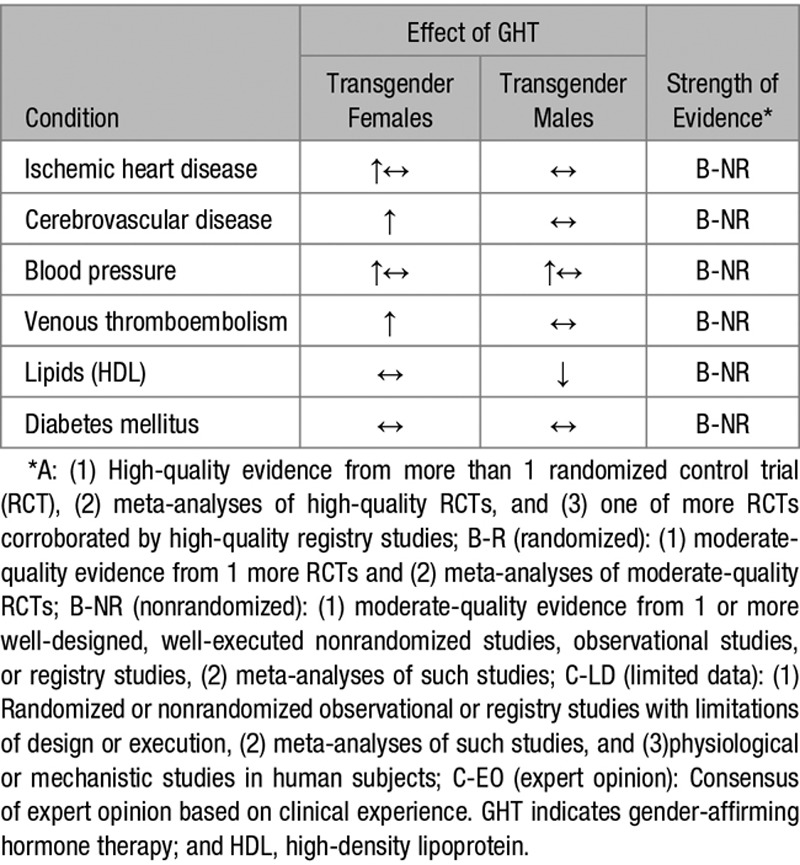
Summary of the Effects of GHT in the Vascular Health of Transgender Females and Males

## Sources of Funding

This work was supported by the British Heart Foundation (Center of Research Excellence Awards RE/13/5/30177 and RE/18/6/34217).

## Disclosures

None.
